# Long-Chain Molecules with Agro-Bioactivities and Their Applications

**DOI:** 10.3390/molecules28155880

**Published:** 2023-08-04

**Authors:** Fahong Yin, Zhaohai Qin

**Affiliations:** College of Science, China Agricultural University, Beijing 100193, China; yinfahong0206@163.com

**Keywords:** long-chain molecules, agro-bioactivities, pesticides

## Abstract

Long-chain molecules play a vital role in agricultural production and find extensive use as fungicides, insecticides, acaricides, herbicides, and plant growth regulators. This review article specifically addresses the agricultural biological activities and applications of long-chain molecules. The utilization of long-chain molecules in the development of pesticides is an appealing avenue for designing novel pesticide compounds. By offering valuable insights, this article serves as a useful reference for the design of new long-chain molecules for pesticide applications.

## 1. Introduction

**Figure 1:**
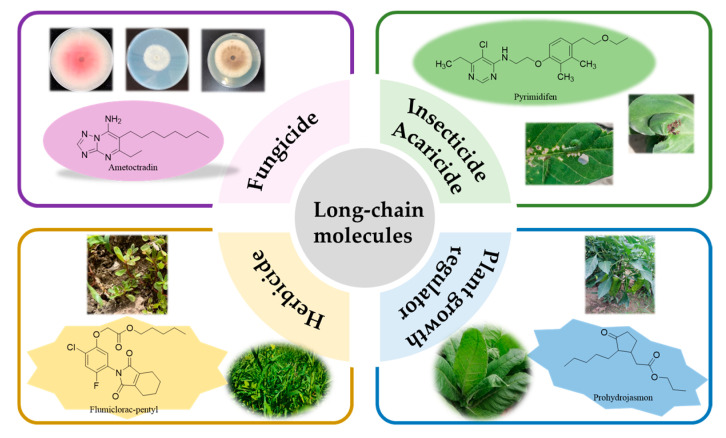
Classification of long-chain molecules in this article.

Plant pests, diseases, and weeds are significant challenges in agriculture, as they can lead to decreased crop yields and pose threats to food safety. While resistant crop varieties and other biological strategies have been employed to combat these issues, pesticides remain the primary means of control [1,2,3]. Among the various types of pesticides, long-chain molecules—both natural and synthetic—are widely used for their efficacy as fungicides, insecticides, acaricides, herbicides, and plant growth regulators (Figure 1). For the purposes of this review, we define long-chain molecules as compounds with five or more atoms (C, O, and S) in their chains. We exclude long-chain molecules containing nitrogen atoms due to their unique effects on the biological activity of the compounds, such as their tendency to form hydrogen bonds. In this article, we primarily focus on the agricultural biological activities and applications of long-chain molecules.

## 2. Long-Chain Molecules Exist Widely in Nature

Long-chain molecules are prevalent in nature and are involved in vital physiological processes in living organisms. These molecules exhibit diverse biosynthetic pathways and possess remarkable biological properties. This section provides a concise overview of various natural long-chain molecules, focusing on their biosynthetic pathways and biological activities.

**Figure 2:**
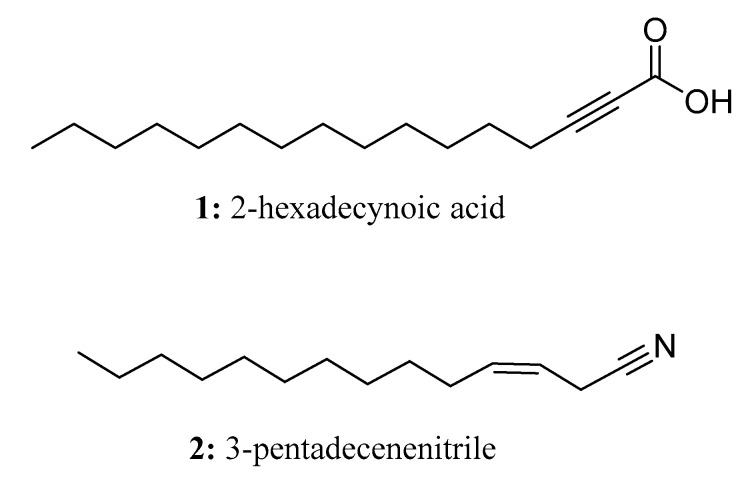
The structures of compounds **1** and **2**.

In nature, fatty acids (FAs) have wide-ranging functions and are found in abundance. They play important roles in energy storage, structural organization, and signaling processes [4,5]. Plants utilize a complex enzymatic system called the plastid-localized fatty acid synthase complex to synthesize fatty acids. This complex acts on acyl-intermediates that are attached to acyl carrier proteins (ACPs). The synthesis process starts with acetyl-CoA, and carbon dioxide is released through the condensation reaction between acetyl-CoA and malonyl-ACP. Subsequent reactions lead to the formation of carbon-expanded, saturated hydrocarbon chains from the intermediate 3-ketone. As a result of the fatty acid synthase cycles, saturated C16 and C18 fatty acids are produced [6,7]. It is noteworthy that many long-chain fatty acids exhibit significant biological activity. For instance, scleropyric acid demonstrates anti-plasmodial activity, 2-hexadecynoic acid (**1**) (Figure 2) and 2-alkynoic fatty acid have been found to exhibit antibacterial activity against mycobacterium tuberculosis, and linoleic acid exhibits antifungal activity [8,9,10]. Furthermore, oleic acid has been shown to possess antifungal effects against *Rhizoctonia solani*, *Pythium ultimum*, *Pyrenophora avenae*, and *Crinipellis perniciosa* [5,11].

Natural products containing long-chain aliphatic nitriles are produced by two types of bacteria: Gram-positive *Micromonospora echinospora* and Gram-negative *Pseudomonas veronii* [12]. Some of these nitriles have antimicrobial activity. For example, 3-pentadecenenitrile (**2**) (Figure 2) has been found to be effective against bacteria such as *Bacillus subtilis*, *Micrococcus luteus*, and particularly *Staphylococcus aureus*, including drug-resistant strains. Nitriles are synthesized from fatty acids, which are first converted into amides and then dehydrated. The process of chain elongation and dehydration is variable during fatty acid biosynthesis, resulting in the formation of unbranched saturated or unsaturated nitriles with an ω-7 double bond. For instance, (Z)-11-octadeconitrile is an example of an unbranched nitrile, while (Z)-13-methyl-tetracylcarbon-3-nitrile is an example of a methyl-branched unsaturated nitrile with the double bond located at C-3.

**Figure 3:**
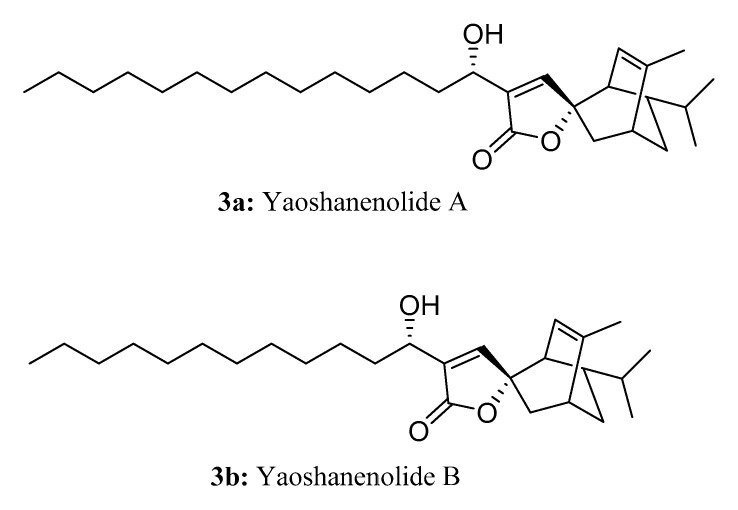
The structures of compounds **3a** and **3b**.

Yaoshanenolides A (**3a**) and B (**3b**) are novel tricyclic spirolactones isolated from the bark of *Machilus yaoshansis* [13]. These compounds contain long linear chains and have exhibited non-selective cytotoxic activity against various human cancer cell lines. Co-occurring obtusilactone A and/or isoobtusilactone A and dihydroisoobtusilactone are believed to be biosynthetic precursors of yaoshanenolides A and B, both of which were isolated from the *Machilus* genus. The biosynthesis of yaoshanenolides A and B involves an enzyme-catalyzed Diels–Alder [4 + 2] cycloaddition between a molecule of the precursors and a molecule of β-phellandrene, which has also been found in *Machilus genus*. This is followed by either simultaneous or sequential allylic hydroxy rearrangement [13,14,15] (Figure 3).

**Figure 4:**
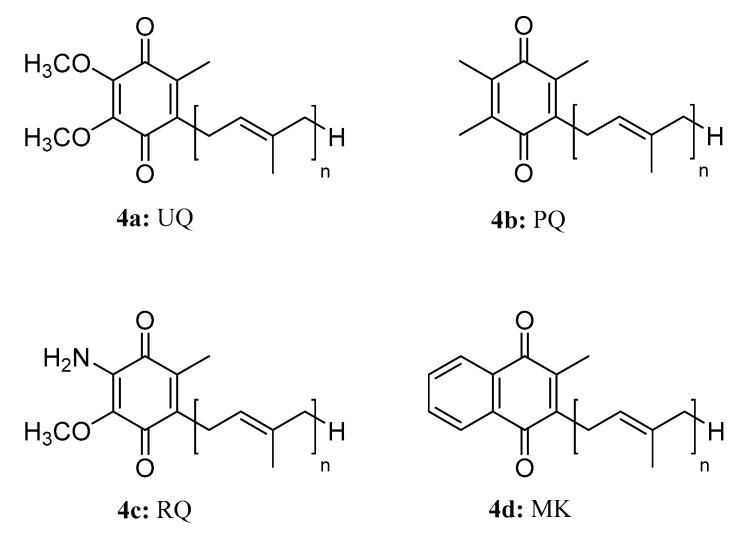
The structures of compounds **4a**–**4d**.

Isoprenoid quinones, which contain a quinone head group and a poly-pentenyl tail of varying length play a crucial role in bioenergetics as electron and proton carriers in the respiratory chain of most organisms [16]. They can be categorized into naphthoquinones and benzoquinones based on the characteristics of their head groups and midpoint redox potentials. Menaquinone (MK, **4d**) belongs to the naphthoquinones, while ubiquinone (UQ, **4a**), plastoquinone (PQ, **4b**), and rhodoquinone (RQ, **4c**) belong to the benzoquinones [17] (Figure 4). The biosynthetic pathway of isoprenoid quinones varies across species. For example, in *Escherichia coli*, the biosynthesis of UQ_8_ (ubiquinone 8) initiates with 4-hydroxybenzoic acid (4-HB) and involves twelve proteins (UbiA to UbiK and UbiX). First, UbiC removes pyruvate from chorismite to form 4-HB [18]. Then, the membrane-bound UbiA prenylates 4-HB using octaprenyl diphosphate as a precursor for the side chain. After prenylation, the UbiD–UbiX system decarboxylates 4-HB [19]. UQ_8_ biosynthesis involves two *O*-methylation reactions catalyzed by the *S*-adenosyl-L-methionine-dependent UbiG protein [20,21]. In this pathway, UbiE catal00yzes the C-methylation reaction, while three class A flavoprotein monooxygenases (FMOs)—UbiH, UbiI, and UbiF—catalyze the hydroxylation of carbon atoms C-1, C-5, and C-6 on the aromatic rings, respectively [22,23].

## 3. Long-Chain Molecules with Agro-Bioactivities

Long-chain molecules exhibit a diverse assay of biological activities and are frequently utilized as fungicides, insecticides, herbicides, and plant growth regulators in agricultural settings. Despite sharing the common feature of a long-chain molecular structure, these compounds operate through a multitude of different mechanisms. Among these fungicides, compounds can be classified based on their mechanism of action, which includes respiratory chain inhibitors, 14α-demethylation inhibitors, and DNA/RNA synthesis inhibitors. Insecticides, on the other hand, include respiratory chain inhibitors, acetylcholinesterase inhibitors, and sodium channel inhibitors. Herbicides, which are made up of long-chain molecules, exhibit different mechanisms of action. For example, acetanilide compounds inhibit the biosynthesis of long-chain fatty acids in plants. Additionally, some compounds inhibit 4-HPPD, resulting in plant albinism and eventually death. In conclusion, long-chain pesticide compounds are not only widely used but also complex. In addition to commercial pesticides, there are many natural products or long-chain derivatives with pesticide activity. In this section, we provide some examples of such compounds.

### 3.1. Phytopathogenic Fungicides Containing a Long Chain

**Figure 5:**
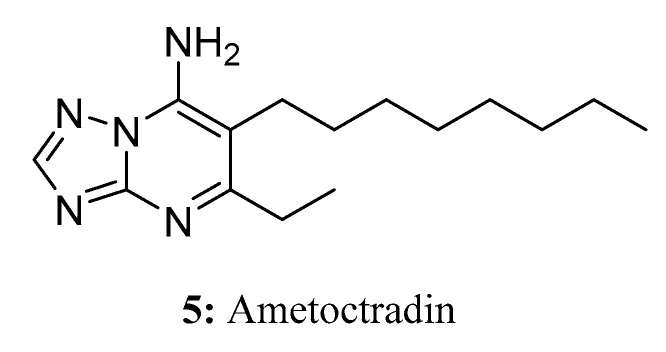
The structure of compound **5**.

Ametoctradin (**5**) [24,25] is a novel oomycete inhibitor with a unique chemical scaffold. Spray applications of ametoctradin, which belongs to the triazolopyrimidine fungicide class, can bring extensive benefits to various special crops. Field trials have demonstrated that ametoctradin is highly selective and effective in preventing late blight and downy mildew (Figure 5).

On the molecular level, ametoctradin functions as an inhibitor of the respiratory *bc*_1_ complex found in oomycete crop pathogens. It achieves this by binding to either the Q_o_-stigmatellin subsite or the Q_i_-site of cytochrome b within complex III of the respiratory electron transport chain, thereby impeding electron transfer. As a consequence of its action, the consumption of oxygen and the levels of intracellular ATP diminish rapidly after ametoctradin treatment in oomycete pathogens. Due to its ability to hinder multiple energy-intensive processes, such as zoospore formation and release, zoosporangia release, germination, and motility, ametoctradin has been designated as a fungicide belonging to the category of QoSI (quinone outside inhibitors) [24,26,27].

**Figure 6:**
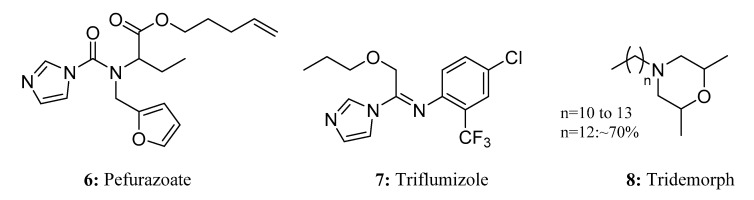
The structures of compounds **6**–**8**.

Pefurazoate (**6**), a novel imidazole compound, is a rice-seed disinfectant. As a fungitoxic agent, it is effective against seed-borne pathogenic fungi such as *Gibbeyella fujikuyoi*, *Pyricularia oryzae*, and *Cochliobolus miyabeanus* [28]. Similar to other azole fungicides, pefurazoate acts as by inhibiting the 14α-demethylation of 24-methylene dihydro lanosterol [28,29]. In addition to its sterilization and disease-control effects, pefurazoate also has a positive effect on promoting seed germination and seedling growth, thereby promoting healthy rice plant development (Figure 6).

Triflumizole (**7**) is one of the imidazole fungicides [30]. This fungicide is known for its high efficiency, low toxicity, and low residual properties. It contains fluorimidazole heterocyclic and exhibits both protective and therapeutic effects. Similar to pefurazoate, triflumizole works by inhibiting ergosterol biosynthesis through interference with the demethylation of the ergosterol skeleton at C-14 [31]. Triflumizole is widely used in various fruit and vegetable production areas, such as those for rice, sweet corn, apples, grapes, pears, and cherries, to control the growth and spread of powdery mildew [30,32] (Figure 6).

Tridemorph (**8**) belongs to the morpholine fungicides [33]; it is a systemic fungicide, as it is taken up by the leaves and roots. It acts as an inhibitor of ergosterol biosynthesis, especially through the inhibition of sterol reduction (sterol-Δ^14^-reductase) and isomerization (Δ^8^ to Δ^7^-isomerase) [34]. This fungicide has a broad spectrum of activity and is commonly used for the prevention and control of grain powdery mildew and *Mycosphaerella* spp. in bananas [34,35] (Figure 6).

**Figure 7:**
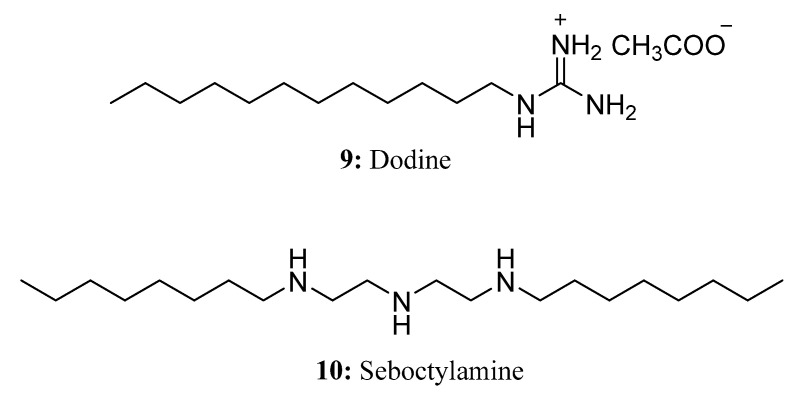
The structures of compounds **9** and **10**.

Dodine (**9**), commonly used as a protective fungicide, contains a guanidyl headgroup and a dodecyl tail [36]. A variety of major mold diseases can be controlled by dodine on fruit trees, vegetables, nuts, ornamental plants, and shade trees [37]. As a dodecyl derivative of guanidinium salt, dodine differs from guanidinium salt because of the presence of a long hydrocarbon chain [38]. It attaches readily to the surfaces of negatively charged microorganisms and can penetrate their membranes, thereby damaging cellular structures [37] (Figure 7).

Seboctylamine (**10**) is a kind of high-efficiency, low-toxicity, and spectral fungicide developed in China [39]. Seboctylamine exhibits a pronounced killing and inhibitory effect on various types of fungi, bacteria, and viruses. Moreover, it demonstrates a potent inhibition effect specifically on the growth of mycelium and the germination of spores [40]. The mechanism of seboctylamine can produce ionization in aqueous solutions; the hydrophilic group part is strongly electropositive, while the adsorption usually contains all kinds of negatively charged bacteria and viruses. It inhibits the multiplication of bacteria and viruses by causing bacterial proteins to coagulate and the polymer to form a film that blocks the ion channels of these microorganisms, causing them to suffocate and die immediately, thus achieving optimal bactericidal effects [39,40,41] (Figure 7).

**Figure 8:**
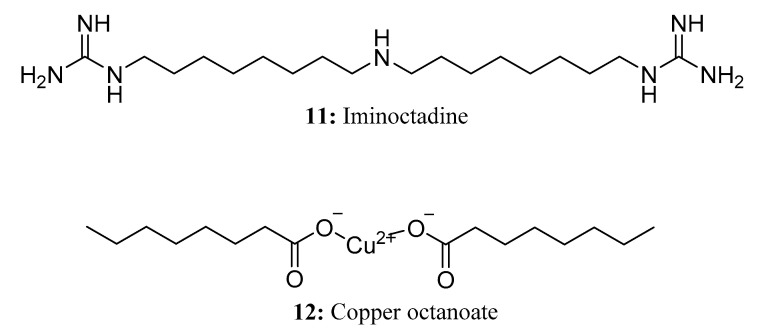
The structures of compounds **11** and **12**.

Iminoctadine (**11**) is a non-systemic aliphatic nitrogen contact fungicide that exhibits preventive effects [42]. It is highly effective against a wide range of fungal diseases caused by ascomycetes, including gray mold, powdery mildew, sclerotinia, and others [43,44]. Iminoctadine affects the biosynthesis of fungal lipids, disrupts the function of fungal cell membranes, and suppresses the formation of appressorium and mycelium through inhibition of spore germination. Iminoctadine tris, on the other hand, is a compound resulting from the combination of iminoctadine with three alkylbenzene sulfonates. Likewise, iminoctadine triacetate refers to the triacetate salt of iminoctadine. This fungicide works by disrupting the membrane function of the pathogen’s cells [45] (Figure 8).

Copper octanoate (**12**) [46,47] is a kind of saturated fatty acid that combines copper ions and caprylic acid, which has the action of contact. Copper octanoate can be used as a fungicide and bactericide for leaf surfaces to control a variety of plant diseases in various crops and landscape plants (Figure 8).

**Figure 9:**
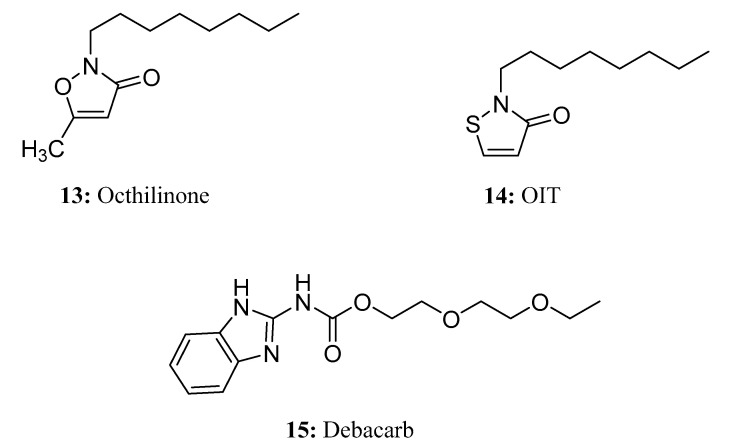
The structures of compounds **13**–**15**.

Octhilinone (**13**) is a DNA/RNA synthesis inhibitor that is effective against apple and pear canker (*Nectria galligena*) as well as other bacterial and fungal diseases (*Ceratocystis* spp.) of top fruits and citrus fruits [48]. Octhilinone is also one of the most frequently used biocides in construction materials as an in-can or film preservative [49]. In addition to being used as a preservative in many industrial applications, including cooling lubricants, sealants, and adhesives, 2-octyl-2H-isothiazol-3-one (OIT, **14**) is a well-known indoor surface fungicide [50] with a strong ability to control mold. Debacarb (**15**) is used via injection for the control of various fungal diseases of trees [51] (Figure 9).

**Figure 10:**
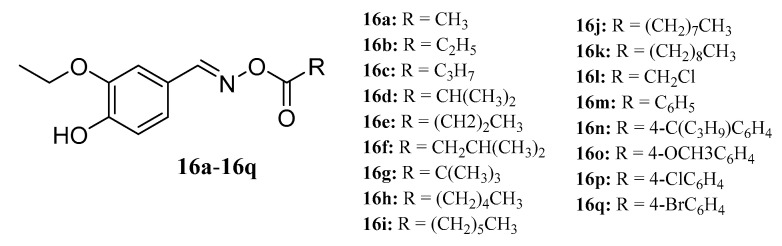
The structures of compounds **16a**–**16q**.

Ahluwalia et al. synthesized a series of oxime esters (**16a**−**16q**) with 3-ethoxy-4-benzaldehyde oxime in the presence of triethylamine and acid chloride. The antibacterial activity of three plant pathogens (*Rhizoctonia bataticola*, *Fusarium udum*, *and Alternaria porii*) was evaluated in vitro. Compounds containing medium-long alkyl chains showed higher activity than those containing long alkyl chains [52] (Figure 10).

**Figure 11:**
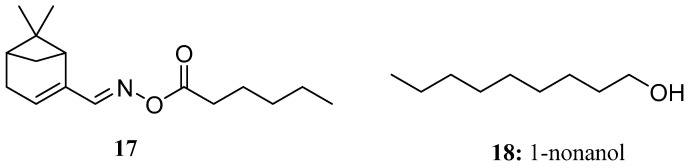
The structures of compounds **17** and **18**.

Compound **17** is a novel myrtenal oxime ester molecule designed and synthesized by Lin et al. using ketol-acid reductoisomerase as the target enzyme. The results showed that the growth inhibition rate of compound **17** on *Brassica campestris* was 64.2% and that on *Echinochloa crusgalli* L. was 81.8% at 100 μg/mL. Meanwhile, compound **17** exhibited certain in vitro antifungal activity against all tested fungi, such as *Fusarium oxysporum f. cummerinum*, *Physalospora piricola*, *Cercospora arachidicola*, and *Gibberella zeae* [53] (Figure 11).

1-Nonanol (**18**), which is the main component of cereal volatiles, has potential antifungal activity against *Aspergillus flavus*. The damaging effect of 1-nonanol on the growth of *Aspergillus flavus* is manifested by intracellular electrolyte leakage; reduced succinate dehydrogenase, mitochondrial dehydrogenase, and ATPase activities; and reactive oxygen species accumulation. We speculated that 1-nonanol can damage the membrane integrity and mitochondrial function of *Aspergillus flavus* and may lead to the apoptosis of *Aspergillus flavus* [54] (Figure 11).

**Figure 12:**
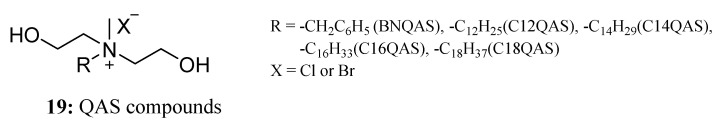
The structure of QAS compounds.

Liu et al. prepared five quaternary ammonium salt (QAS) compounds (**19**) (R = -benzyl (chloride, BNQAS), -dodecyl (C12QAS), -tetradecyl (C14QAS), hexadecyl (C16QAS), and -octadecyl (C18QAS)), and their antifungal properties were tested (Figure 12). The results indicated that C12QAS is effective against several apple fungi, including *Cytospora mandshuria*, *Botryosphaeria ribis*, *Physalospora piricola*, and *Glomerella Cingulata*. The antifungal activity of QAS is mainly related to the introduction of a long-chain alkyl in the molecule. The long alkyl chains in QAS can change the geometry of QAS, which can help the two flexible hydroxyl groups of QAS cross the cell membrane and enter the cell to cause damage [55].

**Figure 13:**
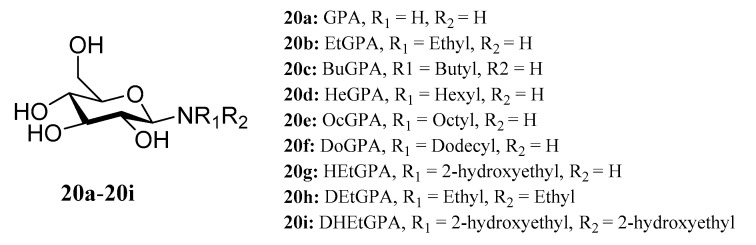
The structures of compounds **20a**–**20i**.

Muhizi et al. synthesized different *N*-alkyl-*β*-d-glucosylamines (**20a**−**20i**) and evaluated their antifungal activities. The results showed that these compounds have different biological activities and that the antifungal activity of glucosylamines increases with the length of the alkyl chain. DoGPA was more bioactive against all target strains than other N-alkyl glucosamines and could be used to inhibit *Fusarium proliferatum* [56] (Figure 13).

### 3.2. Insecticides/Acaricides Containing a Long Chain

**Figure 14:**
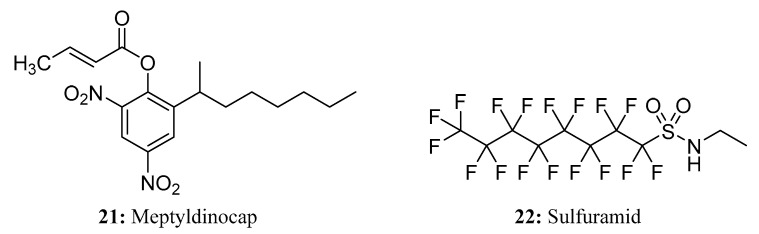
The structures of compounds **21** and **22**.

Dinocap is a mixture of six isomers of dinitrooctylphenyl crotonate (2,4-DNOPC), including ortho and para methylheptyl, ethylhexyl, and propylpentyl crotonate isomers [57]. It is a contact fungicide and acaricide that has been used to control mites in apple crops and powdery mildew in orchard fruits, vegetables, and ornamental crops [58]. The new meptyldinocap (**21**) is an improved version of the single 2,4-DNOPC methylheptyl isomer with a better toxicological profile compared to the old dinocap. It is a non-inhalant acaricide and a powdery mildew (*Erysiphe necator*) fungicide that shows both protectant and post-infection activity [57] (Figure 14).

Sulfuramid (**22**) is a potential bait agent that belongs to a novel class of insecticides known as fluoroaliphatic sulfonamides [59]. It acts as a delayed-acting insecticide by uncoupling oxidative phosphorylation and exhibits chronic toxicity. When insects consume sulfuramid, it acts as a stomach poison, inhibiting their energy metabolism. Sulfuramid has proven effective in controlling leaf-cutting ants (*Atta* spp. and *Acromyrmex* spp.) in eucalyptus and pine plantations [60] (Figure 14).

**Figure 15:**
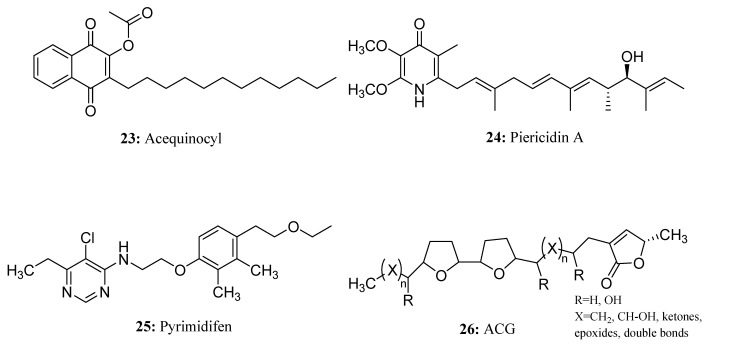
The structures of compounds **23**–**26**.

Acequinocyl (**23**) is a commercially available acaricide that belongs to the naphthoquinone analog group. Acequinocyl acts by inhibiting mitochondrial respiratory complex III. Its deacetylated metabolite contains free hydroxyl groups, which function as ubiquitin analogs, and it is a powerful inhibitor of the Q_o_ center [61]. Acequinocyl is commonly used to control various herbivorous mites, including *Tetranychus urticae* [62]. Acequinocyl exhibits a stronger killing effect on immature spider mites, while its toxicity to mammals is relatively low. Additionally, it has a shorter persistence in the environment [63] (Figure 15).

Piericidins are a group of compounds isolated from actinomyces [64], particularly from the genus *Streptomyces*. One of these compounds, called Piericidin A (**24**), is a natural insecticide that is highly active against lepidoptera larvae. It was first isolated in the 1960s from *Streptomyces mobaraensis* [65,66]. Piericidin A has a similar structure to coenzyme Q, with a head group that resembles ubiquinone and a nitrogen atom in place of one of the carbonyl groups. The hydrophobic tail of Piericidin A contains an isoprenoid group with a hydroxyl group at its end [65]. Piericidin A acts as an antagonist to coenzyme Q, inhibiting the activity of complex I [64] (Figure 15).

Pyrimidifen (**25**) is a potent insecticide that has proven effective in combatting various mite species that pose a threat to fruits and vegetables. It exhibits efficacy against all life stages of spider mites and is also capable of managing the population of diamondback moths (*Plutella xylostella* L.). Pyrimidifen operates through the same mechanism of action as other pyrimidine acaricides: inhibiting the electron transfer on complex Ι [67,68] (Figure 15).

**Figure 16:**
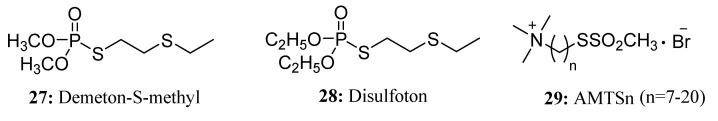
The structures of compounds **27**–**29**.

The main structural feature of ACG (**26**) is an alkyl chain containing a methyl γ-lactone ring at the end, 0~3 tetrahydrofuran rings on the alkyl chain, and a certain number of oxygen-containing functional groups (such as hydroxyl, acetoxy, ketone, epoxide, etc.) or a double bond; the number of carbon atoms is 35 or 37 [69]. According to the number and spatial arrangement of THF in ACG, ACG can be divided into various types, such as mono-THF acetogenins, adjacent bis-THF acetogenins, non-adjacent bis-THF acetogenins, non-THF acetogenins, and so on. Due to the strong inhibitory effect of ACG on the mitochondrial respiratory chain complex Ι of cells, most ACGs have good insecticidal effects, and adjacent bis-THF acetogenins have the most obvious insecticidal effect [70,71] (Figure 15).Demeton-*S*-methyl (**27**) is a widely used systemic organophosphorus insecticide with a certain toxicity that is used to control aphids and red spiders in various crops. Demeton-*S*-methyl irreversibly inhibits cholinesterases by phosphorylating their catalytic serine [72,73] (Figure 16).

Disulfoton (**28**), a kind of organophosphorus pesticide, is a systemic insecticide and acaricide [74,75,76,77] that is normally applied as granules with the seed at sowing [78]. Disulfoton has been extensively used across a wide range of crops to manage sap-feeding insects, including aphids, mites, and thrips [76]. Its mode of action involves inhibiting the enzyme acetylcholinesterase (AChE) [77,79], which leads to the accumulation of acetylcholine in nerve endings in both the peripheral and central nervous systems. Consequently, disulfoton poses a high level of toxicity to humans [80,81,82]. The World Health Organization (WHO) has classified disulfoton as “extremely hazardous” (Figure 16).

AMTSn (**29**), a small molecule containing methanethiosulfonate, was developed by Pang as an inhibitor of AChE activity. It was found to irreversibly inhibit 99% of the AChE derived from the green insect aphid (*Schizaphis graminum*) at a concentration of 6.0 mM. Interestingly, there was no measurable inhibition of human AChE. Reactivation studies using β-mercaptoethanol confirmed that this irreversible inhibition occurs by binding AMTSn to a unique Cys residue found at the active site of AChE in aphids and other insects but not in mammals. This discovery suggests that targeting this specific Cys residue could potentially be used to develop insect-selective insecticides [83,84,85,86] (Figure 16).

**Figure 17:**
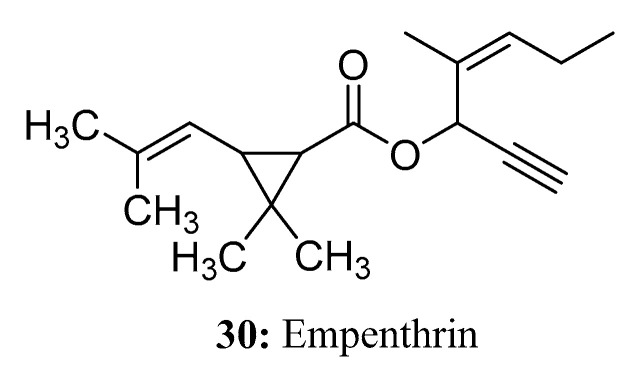
The structure of compound **30**.

Empenthrin (**30**) is a highly volatile synthetic pyrethroid with potent insecticidal activity against houseflies and textile pests and a low toxicity to mammals [87,88]. The efficacy of empenthrin is primarily attributed to the unique structure of its alcoholic part and its high vapor pressure. This pyrethroid is formed through the combination of synthetic ethynyl alcohol and chrysanthemic acid, in part mimicking the rethrolone skeleton found in natural pyrethroids [89] (Figure 17).

**Figure 18:**
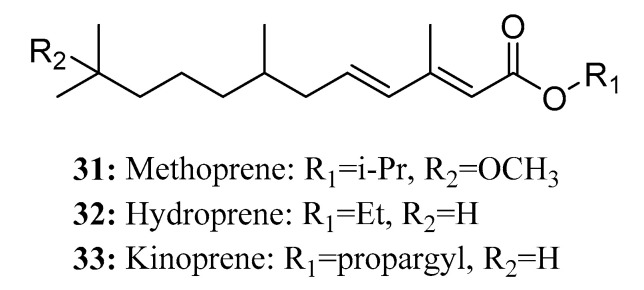
The structures of compounds **31**–**33**.

Juvenile hormone (JH) analogs, namely methoprene (**31**), hydroprene (**32**), and kinoprene (**33**), are widely used in pest control due to their non-toxicity toward vertebrates and other non-target organisms [90] as well as their rapid degradation after application (Figure 18).

Methoprene, the first synthetic insect juvenile hormone analog, is known for its efficacy in pest control. In particular, *S*-methoprene is a highly effective form of this compound. Acting as a synthetic insect juvenile hormone analog, S-methoprene operates through a mechanism similar to that of natural juvenile hormones. It interferes with the normal growth and development process of insects, causing diapause to occur, and disrupts the development of larvae to adults, thereby inhibiting the reproduction of pests [91,92]. Methoprene has been proven to be particularly effective against harmful species of Diptera and Coleoptera [93,94]. Furthermore, it has been applied in the prevention and control of ants and animal fleas [95,96].

Hydroprene is effective against certain lepidopteran pests by imitating the actions of naturally occurring JHs [97]. Application of hydroprene at the larval stage limits and suppresses normal larval development. Larvae treated with hydroprene either fail to reach adulthood or become abnormally sterile adults [98]. As an active ingredient, Kinoprene exhibits particularly high activity against Homopteran insects.

In addition, Mori et al. prepared and bioassayed twenty-six juvenile hormone analogs with varying molecular chain lengths using allatectomized fourth instar larvae of *Bombyx mori* L. Their findings revealed that among methyl or ethyl esters, the optimal chain length for the high juvenile hormone activity on the silkworm is 17 atoms [99]. Additionally, Kisida et al. reported the discovery of thiolcarbamates and their derivatives, which showed strong larvicidal activities against the larvae of *C. pipiens* and *A. aegypti,* similar to JHs [100].

**Figure 19:**
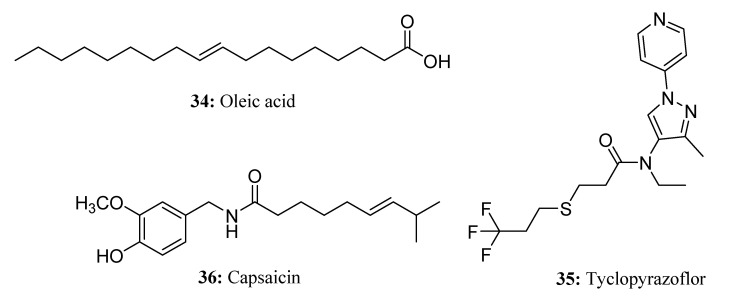
The structures of compounds **34**–**36**.

Oleic acid (**34**) can destroy insect cuticles, and its potassium and sodium salts can be used as an insecticide against soft-bodied pests (aphids, whiteflies, and spider mites) on vegetables, fruits, and ornamentals, as well as a fungicide against powdery mildew, while oleic acid can also be used for weed control on uncultivated land [101,102,103]. In addition, oleic esters are also widely used in the field of pesticides. For example, methyl oleate is usually used as emulsifier and auxiliary for pesticides, which can increase the degree of dispersion of the agent and improve the utilization rate of the pesticide [104,105,106], while ethyl oleate can be used for the preparation of fungicides, acaricides [107,108], etc., which have the advantages of high efficiency, no residue, a low production cost, environmental protection, and excellent comprehensive performance (Figure 19).

Tyclopyrazoflor (**35**) is a pyridyl-pyrazole insecticide with a good killing effect against sap-feeding insects. This compound belongs to a novel category of chemicals that effectively manage infestations caused by *Myzus persicae* and sweet potato whitefly crawlers [109,110,111,112]. Unlike conventional ryanodine receptor insecticides, Tyclopyrazoflor exhibits a distinctive structure characterized by a unique amide group (Figure 19). Capsaicin (**36**) is a natural alkaloid obtained from the mature fruit of the capsicum [113]. Studies have shown that capsaicin has a stimulating effect in various species, can function as a repellent, and can also influence the egg-laying decisions of various insects [113,114,115]. As a botanical pesticide, capsaicin exhibits several desirable characteristics, including high effectiveness, prolonged duration, degradability, and non-toxicity (Figure 19).

**Figure 20:**
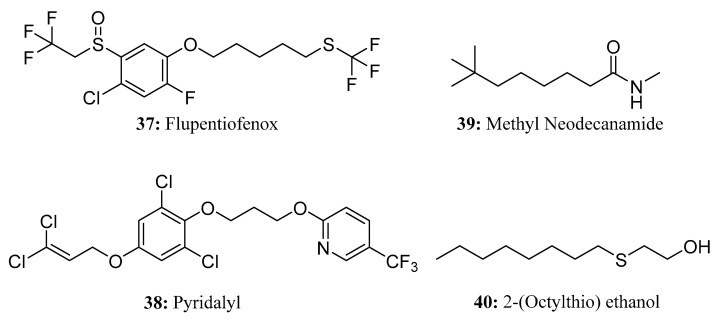
The structures of compounds **37**–**40**.

Flupentiofenox (**37**) is a new trifluoromethyl thioether acaricide. It is a racemate that contains chiral sulfur atoms. It has a novel structure with an unknown mechanism of action. Notably, flupentiofenox demonstrates excellent activity against adult tetranychus and larvae of the brown planthopper, even at low concentrations. As such, it can be used to control harmful arthropods, including mites on fruits and vegetables and planthoppers on rice [116] (Figure 20).

Pyridalyl (**38**) is a highly efficient and low-toxicity insecticide with excellent control efficacy against Lepidoptera and Thysanoptera pests [117]. Due to its unique chemical structure and characteristic insecticidal profile [118], it has no cross-resistance with various currently used insecticides in *Plutella xylostella* or *Heliothis virescens*, inferring that it may have a new mechanism of action [119,120,121] (Figure 20).

Methyl neodecanamide(MNDA, **39**) is an isomeric distribution of secondary amides that has shown efficacy as an insect repellent [122,123]. MNDA can be used as insect repellent for cockroaches, mosquitoes, moths, flies, fleas, ants, lice, spiders, ticks, and mites; for this reason, it is a useful ingredient in household cleaners [122,123] (Figure 20).

2-(Octylthio)ethanol (**40**) [124] is a new microtoxic (nearly non-toxic) and highly effective insect repellent that exhibits a strong ability to repel mosquitoes, flies, reptiles, ants, bedbugs, and other insects (Figure 20).

**Figure 21:**
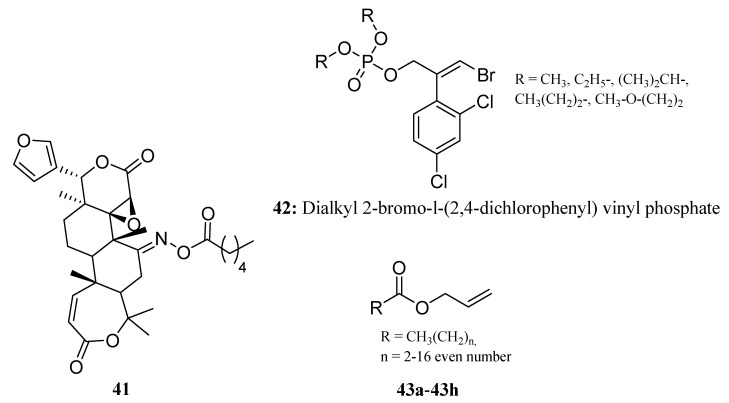
The structures of compounds **41**–**43**.

Compound **41** (Figure 21) belongs to a series of C7-oxime ester derivatives (*n* = 0~6,8,9,14,16) of obacunone prepared by Xiang Yu; its insecticidal activity against the pre-third-instar larvae of oriental armyworm (*Mythimna separata* Walker) was evaluated. A comparison of compound **41** (*n* = 4) with the precursor obacunone showed that compound **41** (*n* = 4) displayed greater insecticidal activity with final mortality rates greater than 60% [125].

Fulde reported a series of dialkyl 2-bromo-l-(2,4-dichlorophenyl) vinyl phosphates (**42**) that exhibited biological activity against the house fly (*Musca domestica* L.). The toxicity of these compounds to flies was found to decrease with an increase in the length of the alkyl chain. The methyl ester was identified as the most active compound, while the 2-methoxyethyl ester was the least active [126]. This suggests that the biological activity of these compounds is not solely dependent on the length of the alkyl chain (Figure 21).

Escriba et al. synthesized a group of allyl esters of fatty acids (**43a**–**43h**) (Figure 21) from glycerol. These compounds showed ovicidal activity against codling moth eggs, and the alkyl chain length was inversely related to the ovicidal activity of the allyl esters. Notably, the two compounds with longer alkyl chains showed significantly lower ovicidal activity compared to the other compounds [127].

**Figure 22:**
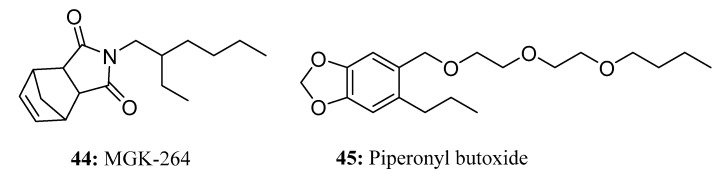
The structures of compounds **44** and **45**.

MGK-264 (**44**) [128,129,130] has been shown to enhance the insecticidal activity of pyrethrin, pyrethroid, and carbamate insecticides. When used in conjunction with pyrethrin or allethrin, this synergist is especially effective in preventing and controlling cockroach infestations. Importantly, MGK-264 does not possess any inherent toxicity in insects (Figure 22).

The semi-synthetic compound piperonyl butoxide (**45**) (Figure 22) is a methylenedioxyphenyl (MDP) compound derived from natural safrole extracted from sassafras oil [131,132]. By inhibiting insect cytochrome P450 (CYP) and esterase enzymes, piperonyl butoxide enhances the insecticidal activity of natural and synthetic pyrethroids and other insecticides [133]. Piperonyl butoxide exhibits characteristics such as prolonging the drug-holding time, broadening the insecticidal spectrum, reducing pesticide dosages, lowering costs, and being safe and non-toxic.Insect pheromones and their analogs are a very large family containing a vast number of long-chain molecules. These pheromones serve various highly specific functions, such as attracting, stimulating, facilitating or inhibiting feeding, promoting egg laying, facilitating mating, organizing assembly, alarming, and defending. Due to the wide variety of insect pheromones (both commercialized and analogues) under development, they were not reviewed in this paper.

### 3.3. Herbicides Containing a Long Chain

**Figure 23:**
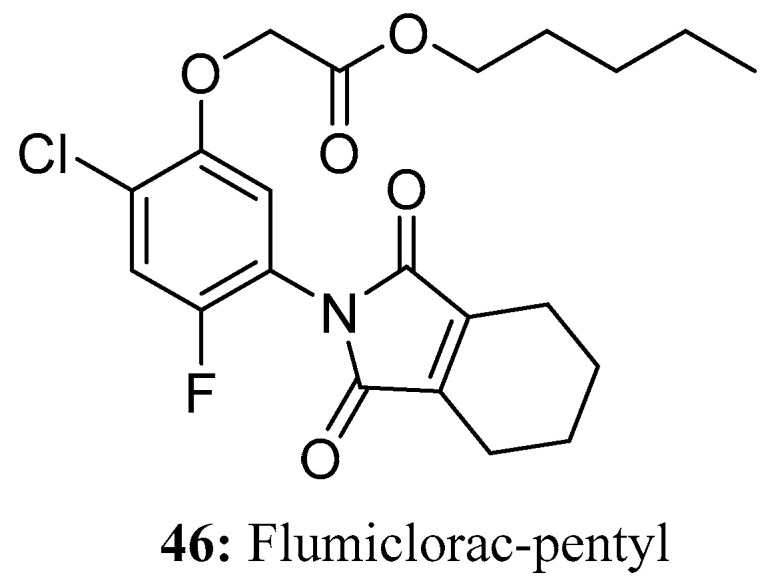
The structure of compound **46**.

Flumiclorac-pentyl (**46**) is a post-emergence herbicide that exhibits selective efficacy against broadleaf weeds in soybeans. Problematic weeds such as velvetleaf, prickly sida, jimsonweed, and common lambsquarters can be effectively controlled with flumiclorac-pentyl [134]. The herbicide is absorbed by the leaves and sheaths of plants, transmitted via the phloem, and accumulates in the meristem region. Flumiclorac-pentyl demonstrates resilience against acetyl-CoA carboxylase by hindering fatty acid synthesis, disrupting cellular growth and division, and impairing lipid structures like membrane systems, ultimately inducing plant death. Remarkably, flumiclorac-pentyl stands out among aromatic oxyphenoxypropionic acid herbicides due to its exceptional safety profile for rice cultivation (Figure 23).

**Figure 24:**
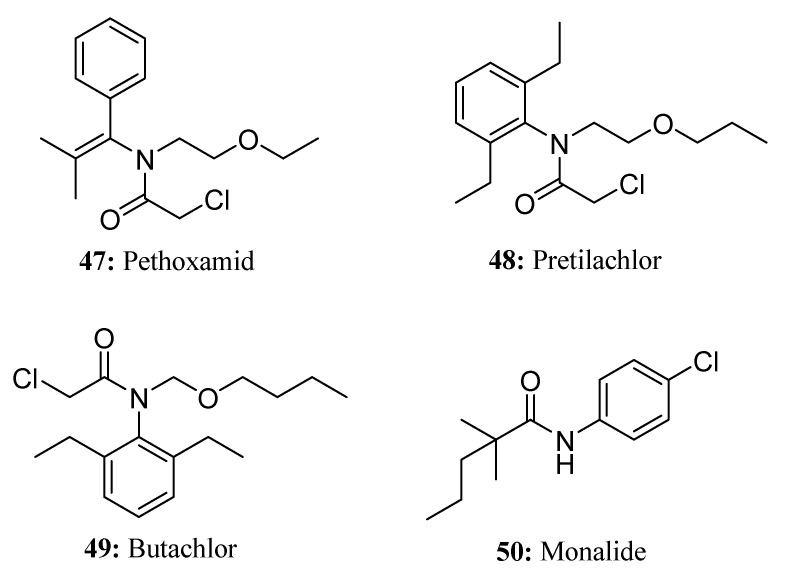
The structures of compounds **47**–**50**.

Pethoxamid (**47**) (Figure 24) is a novel chloroacetamide herbicide that was developed by Tokuyama in Japan through modification and repeated screening of the chemical structure and biological activity of the rice field herbicide thenylchlor. Pethoxamid is typically applied to the soil as a pre-plant, pre-plant-incorporated, or pre-emergence herbicide [135]. It has shown effective weed control against broadleaved weeds such as redroot pigweed (*Amaranthus retroflexus* L.), common lambsquarters (*Chenopodium album* L.), and ladysthumb (*Polygonum persicaria* L.) as well as annual grasses that include species from the foxtail species (*Setaria* spp.) and large crabgrass (*Digitaria* spp.) [136,137]. Similar to pretilachlor, pethoxamid inhibits the formation of very-long-chain fatty acids in responsive weeds and is generally used to control weeds before they emerge [137,138].

Pretilachlor (**48**) is a selective pre- or post-emergence herbicide that belongs to the chloroacetanilide class (Figure 24). It is widely used to control broadleaved weeds, various grasses, and sedges in transplanted and directly seeded paddy fields [139,140,141,142,143,144,145,146,147,148]. Pretilachlor works by inhibiting the synthesis of very-long-chain fatty acids (VLCFAs) and cell division.

Butachlor (**49**) is one of the widely and extensively used herbicides that is primarily used for controlling grass weeds and various broadleaved weeds in crops such as wheat, rice, and other cereal crops [149,150] (Figure 24). It is a chloroacetamide herbicide that inhibits early plant development by inhibiting the biosynthesis of long-chain fatty acids in microsomes [151,152].

Monalide (**50**), which belongs to the class of aniline herbicides, is an important selective herbicide for controlling weeds in vegetable crops. It is applied after the emergence of both weeds and crops [153,154] (Figure 24).

**Figure 25:**
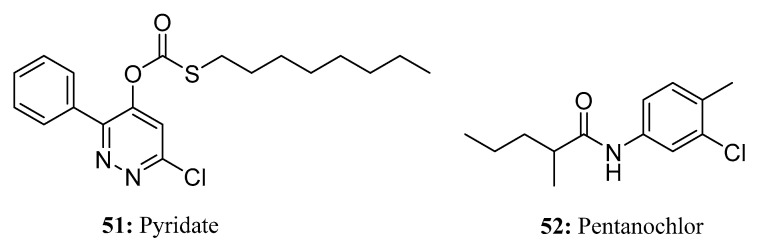
The structures of compounds **51** and **52**.

Pyridate (**51**) is a common single-dose rice field herbicide that belongs to the thiocarbamate herbicides and is a selective post-seedling herbicide (Figure 25). It is suitable for controlling broadleaf weeds of wheat, rice, corn, and other cereal crops, especially for porcine amaranth and some gramineous weeds. It works by blocking the weed’s photosystem II process, leading to rapid degradation of the weed’s cell walls [155].

Pentanochlor (**52**) is a selective, contact herbicide that is absorbed through leaves [156] (Figure 25). Used for selective weeding of carrot, celeriac, celery, fennel, parsley, parsnips, and other plants before and after seeding as well as for the pre-seeding of tomato and some flower crops.

**Figure 26:**
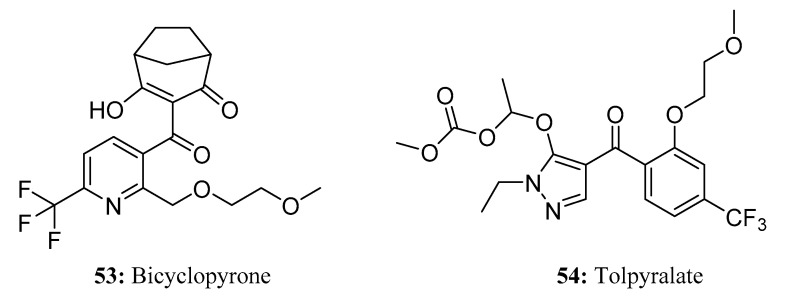
The structures of compounds **53** and **54**.

Bicyclopyrone (**53**) is a highly effective broad-spectrum herbicide that demonstrates exceptional selectivity for use in corn, sugar beet, and grain fields (Figure 26). As a member of the 4-hydroxyphenylpyruvate dioxygenase (4-HPPD) inhibitor herbicide family, bicyclopyrone is responsible for inhibiting the activity of 4-HPPD, a non-heme iron (II)-dependent dioxygenase. Consequently, the biosynthesis of carotenoids is impeded, leading to albinism in the plant meristem and ultimately causing plant death [157,158,159,160,161,162,163]. The application of bicyclopyrone is very flexible; it can be used from pre-sowing to post-emergence, and it also works well in different environmental conditions and different planting methods.

Tolpyralate (**54**) is a novel selective herbicide for controlling weeds in corn crops. It demonstrates effective control over a broad spectrum of grasses and broadleaves while exhibiting excellent selectivity for corn [164] (Figure 26). Tolpyralate is a new 4-HPPD-inhibitor that belongs to the benzoylpyrazole family that affects sensitive weeds by interrupting the biosynthesis of plastoquinone and tocopherol and stopping the synthesis of carotenoid pigments [163,165]. In comparison to other herbicides of the same class, tolpyralate delivers comparable or superior weed control in annual broadleaves and grasses [166,167].

**Figure 27:**
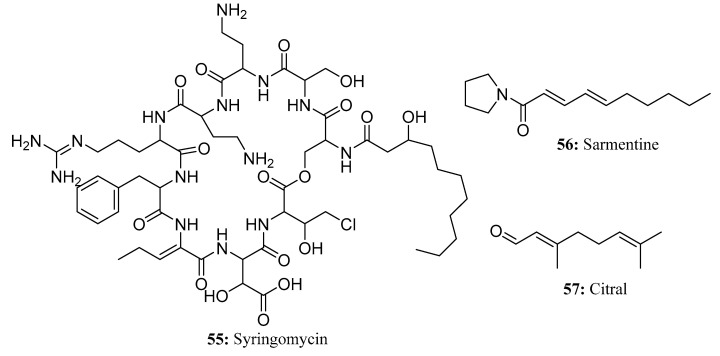
The structures of compounds **55**–**57**.

Syringomycin (**55**), which is derived from *Pseudomonas syringae*, is a large amphiphilic molecule with a polar peptide head and a hydrophobic 3-hydroxy fatty acid tail of variable length (from C10 to C14) that binds to N-terminal serine residues via an amide bond [168] (Figure 27). The mode of action of syringomycin is assembly into macromolecules and insertion into the cell membrane to form pores that can freely permeate cations, resulting in the rapid necrosis of plant tissues [169].

Sarmentine (**56**) is a natural product from *Piper longum* L. that has been patented as an herbicide (Figure 27). Sarmentine destroys the plant cuticle, leading to cell membrane damage followed by rapid drying and eventually tissue death [168].

Citral (**57**) is a diterpene component in many plant essential oils that also has been patented as an herbicide (Figure 27). Citral vapor disrupts microtubule polymerization in *Arabidopsis* seedlings [170], but it may have a new target for disrupting mitosis because citral affects microtubule polymerization differently than other mitotic inhibitors used as herbicides [168].

**Figure 28:**
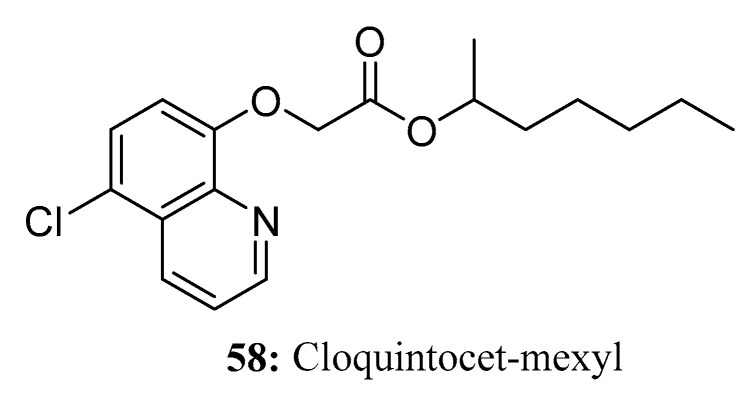
The structure of compound **58**.

Cloquintocet-mexyl (CLM, **58**) is a quinoline-based herbicide safety agent that selectively protects certain crops from herbicide injury without reducing the biological activity of target weeds [171,172,173]. CLM is always used in conjunction with clodinafop-propargyl for the effective control of isoproturon-resistant small-seed canary grass biotypes (*Phalaris minor Retz.*) as well as other broad-leaved weeds (*Triticum aestivum*) [174] while protecting crops from herbicide damage (Figure 28).

### 3.4. Plant Growth Regulators Containing a Long Chain

Plant growth regulators are defined as natural or chemically synthesized substances that play a role in plant development or metabolism.

**Figure 29:**
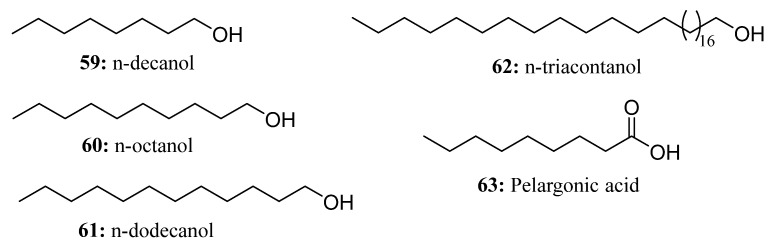
The structures of compounds **59**–**63**.

In 1964, C6-C18 high-carbon primary alcohol was identified as a promising tobacco bud suppressor. Extensive research conducted by tobacco chemists and growers from various countries revealed that n-decanol (**59**) exhibited the most effective suppression, followed by n-octanol (**60**) and n-dodecanol (**61**). Due to its excellent biodegradability and composition of only carbon, hydrogen, and oxygen, high-carbon primary alcohol has gained significant popularity within the international tobacco industry for its non-toxic and non-residual characteristics [175,176] (Figure 29).

TRIA (**62**), a primary saturated alcohol, is classified as a plant growth regulator found in the epicuticular waxes of upper plant surfaces that stimulates physiological and biochemical processes in many crops [177,178] (Figure 29). Relatively low concentrations of triacontanol promote the growth of most crops, including rice (*O. sativa* L.) and maize (*Zea Mays* L.) [179,180]. Currently, TRIA has been used to improve plant tolerance to abiotic stresses such as low temperature, drought, salt stress, and heavy metals [179,181,182]. It is noteworthy that under abiotic stress, exogenous application of TRIA can promote plant growth, increase photosynthetic pigment content, and increase compatible osmolyte accumulation [183,184]. TRIA can also reduce stress by regulating the expression of certain genes [182,185].

Pelargonic acid (**63**) is a fatty acid widely found in nature that occurs not only in animals and plants but also in volatile organic compounds in soil [186,187] (Figure 29). It can be used as a blossom-thinning agent to prevent alternate-year cropping of apples [188]. Pelargonic acid, in the form of its salts and formulations, is also utilized as a non-selective herbicide suitable for gardens [186,189]. As a contact non-selective herbicide, it functions by attacking the cell membrane, resulting in cell leakage and the subsequent breakdown of membrane acyl lipids [187,190].

**Figure 30:**
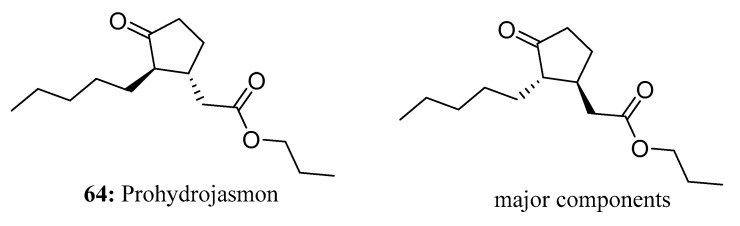
The structure of compound **64**.

Prohydrojasmon (**64**) is a synthetic analog of jasmonic acid (JA) developed as a plant growth regulator [191]. Jasmonic acid (JA) and its derivatives are higher plant growth regulators that are important in regulating physiological processes such as senescence, fruit ripening, coloration, and pigment accumulation. It is believed that these compounds can improve crop quality by increasing anthocyanin, glucosinolate, terpene, and phenolic levels, all of which contribute to the quality of crops [192,193,194,195]. The application of PDJ as a plant growth regulator is mainly focused on fruits, including apples, oranges, mangoes and grapes [191,196,197]. PDJ has similar effects to endogenous JAs and has been shown to affect a variety of physiological processes, including senescence, leaf abscission, fruit ripening, coloration, and pigment accumulation. Other studies have shown that PDJ can also induce bioaccumulation of the above-mentioned secondary compounds, thus improving crop quality [191] (Figure 30).

### 3.5. Long-Chain Molecules with Rodenticidal Activity

**Figure 31:**
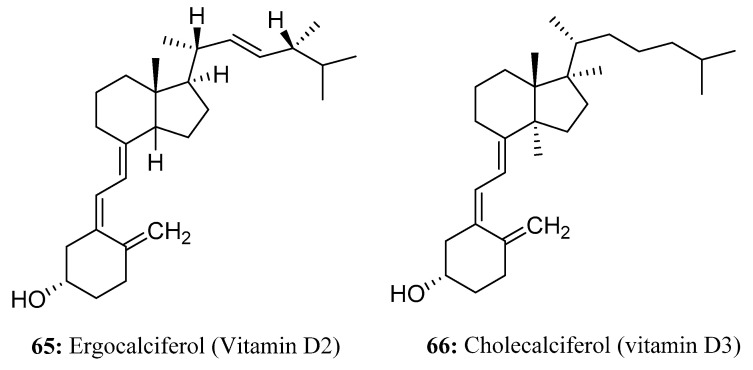
The structures of compounds **65** and **66**.

Vitamin D, also known as calciferol, is a complex lipophilic molecule. Biologically, it is similar to other pseudo-steroids. Ergocalciferol (vitamin D2, **65**) and cholecalciferol (vitamin D3, **66**) are two of the most important and major examples; they are prohormones with very similar chemical structures but different side chains that are involved in calcium and phosphorus metabolism in animals [198]. Ergocalciferol can be used either alone for rodent control or in combination with 0.025% warfarin and 0.005% difenacoum, which are effective combinations for controlling anticoagulant-resistant or -susceptible rats and mice [199]. Ergocalciferol promotes bone calcification and tooth growth in animals and is not harmful to individuals when consumed in small amounts, but if an excessive amount is consumed, calcium in the blood can rise rapidly, leading to tissue calcification and eventual death. Cholecalciferol is also a rodenticidal agent with similar properties (Figure 31).

**Figure 32:**
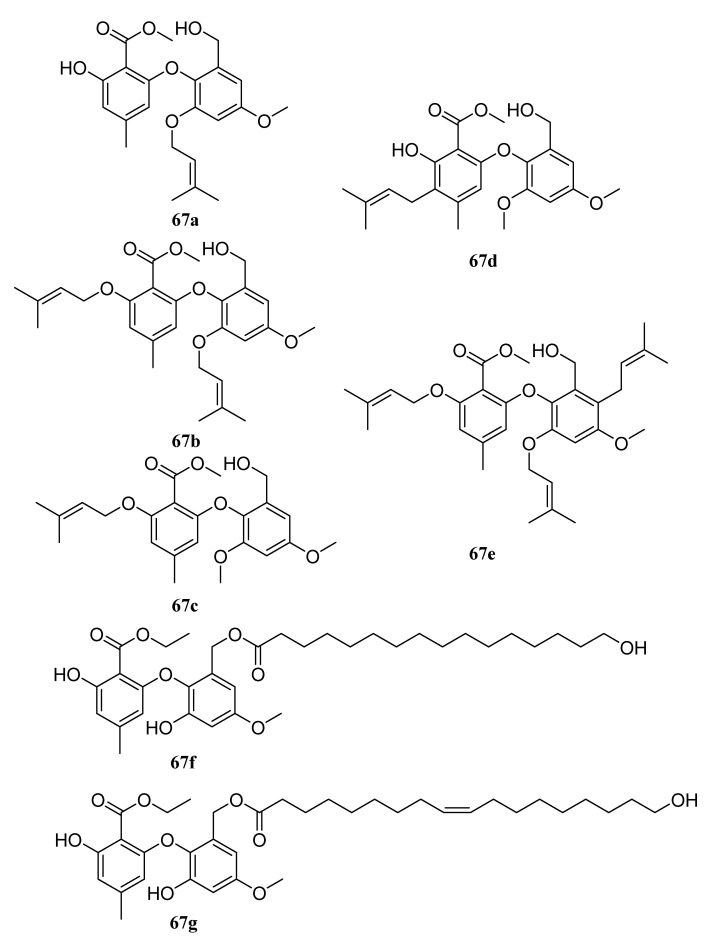
The structures of compounds **67a**–**67g**.

In addition, there are other kinds of long-chain molecules with agro-bioactivities, such as some organic sulfites [200] and non-ionic surfactants [201] like ammonium lauryl sulfate and triethanolamine lauryl sulfate, etc. Natural extracts are also an important source of long-chain molecules with agro-bioactivities [202,203,204]. For example, the crude plant extracts of *Chenopodium ambrosioides*, *Conyza dioscordisis,* and *Convolvulus arvensis* are effective against stored grain pests; the extract components are mainly long-chain fatty acid esters of hexadecanoic, arachidonic, and octadecanoic acids [203]. The hexane (HE) extract of *D. insularis* showed high acaricidal activity against the bovine tick by reducing oviposition and hatching rates [202], which may be related to chemical compounds such as palmitic acid, ethyl hexadecanoate, linolenic acid, and ethyl linolenoate. Zhu et al. purified nine new diphenyl ethers via fermentation using the endophytic fungus *Epicoccum sorghinum* L28. Compounds **67a**–**67e** are long-chain diphenyl ethers, and compounds **67f** and **67g** contain ester segments formed by natural long-chain fatty acids and hydroxyl groups in diphenyl ether benzyl alcohol units (Figure 32). They have strong inhibitory activities against *F. oxysporum* and *C. musa*, which may be a defense tool induced by host chemicals. It can help hosts resist Fusarium and Colletotrichum, which widely infect mangrove plants [205].

## 4. Conclusions and Prospects

In summary, this paper focused on long-chain molecules with pesticide activity, highlighting the importance of the long chain in determining biological activity. The antifungal activity of glucosamine analogs increases with longer alkyl chains, while the ovicidal activity of allyl esters of fatty acids decreases with longer alkyl chains. Long-chain fatty acids with carbon chain lengths of C9~C11 exhibit the best herbicidal activity, indicating variable effects of long-chain tails on biological activity in different molecules. The introduction of long-chains in molecules can alter their geometry and physicochemical properties, offering opportunities for pesticide innovation and agricultural development. Designing long-chain compounds with these properties is an area of interest in pesticide research.

In addition, long-chain molecules exhibit a diverse range of biological activities in agricultural production, making significant contributions to the effective control of agricultural diseases, pests, and weeds while also having a strong economic value. Many agricultural bioactive long-chain molecules possess the advantages of high efficiency and low toxicity. In recent years, several new pesticide products such as seboctylamine, tyclopyrazoflor, and flupentiofenox have been continuously introduced. Furthermore, further research is needed to better understand the mechanism of action and structure–activity relationship of certain long-chain molecules. This will facilitate the development of innovative modifications and compound synthesis, ultimately promoting the wider application of long-chain molecules in agricultural production. The contribution of long chains to the biological activity of these molecules will be a subject that warrants special attention and further research.
